# The consensus on indications, conditioning regimen, and donor selection of allogeneic hematopoietic cell transplantation for hematological diseases in China—recommendations from the Chinese Society of Hematology

**DOI:** 10.1186/s13045-018-0564-x

**Published:** 2018-03-02

**Authors:** Lanping Xu, Hu Chen, Jing Chen, Mingzhe Han, He Huang, Yongrong Lai, Daihong Liu, Qifa Liu, Ting Liu, Ming Jiang, Hanyun Ren, Yongping Song, Zimin Sun, Jianmin Wang, Depei Wu, Daobin Zhou, Ping Zou, Kaiyan Liu, Xiaojun Huang

**Affiliations:** 1 Beijing Key laboratory of Hematopoietic Stem Cell Transplantation, Peking University People’s Hospital & Institute of Hematology, No. 11 Xizhimen South Street, Xicheng District, Beijing, 100044 People’s Republic of China; 2 Affiliated Hospital of The Academy of Military Medical Sciences, Beijing, People’s Republic of China; 3 Shanghai Children’s Medical Center, Shanghai, People’s Republic of China; 4 Chinese Academy of Medical Sciences and Peking Union Medical College, Institute of Hematology and Blood Disease Hospital, Tianjin, People’s Republic of China; 5 First Affiliated Hospital of Zhejiang University, Hangzhou, People’s Republic of China; 6 The First Affiliated Hospital of Guangxi Medical University, Guilin, People’s Republic of China; 7 General Hospital of PLA(People’s Liberation Army of China), Beijing, People’s Republic of China; 8 Nanfang Hospital of Southern Medical University, Guangzhou, People’s Republic of China; 9 West China Hospital, Sichuan University, Chengdu, People’s Republic of China; 10 The First Affiliated Hospital of Xinjiang Medical University, Urumqi, People’s Republic of China; 11 Peking University First Hospital, Beijing, People’s Republic of China; 12 Henan Cancer Hospital, Zhengzhou, People’s Republic of China; 13 Anhui Provincial Hospital, Hefei, People’s Republic of China; 14 Changhai Hospital of Shanghai, Shanghai, People’s Republic of China; 15 The First Affiliated Hospital of Soochow Hospital, Soochow, People’s Republic of China; 16 Peking Union Medical College Hospital, Beijing, People’s Republic of China; 17 Tongji Medical College, Wuhan Union Hospital, Huazhong University of Science and Technology, Wuhan, People’s Republic of China; 18 Peking-Tsinghua Center for Life Sciences, Beijing, People’s Republic of China

**Keywords:** Consensus, Allogeneic hematopoietic transplantation, China, Indication, Conditioning regimen, Donor selection, Standard of care

## Abstract

Allogeneic hematopoietic stem cell transplantation (allo-HSCT) is widely used to treat malignant hematological neoplasms and non-malignant hematological disorders. Approximately, 5000 allo-HSCT procedures are performed in China annually. Substantial progress has been made in haploidentical HSCT (HID-HSCT), pre-transplantation risk stratification, and donor selection in allo-HSCT, especially after the establishment of the “Beijing Protocol” HID-HSCT system. Transplant indications for selected subgroups in low-risk leukemia or severe aplastic anemia (SAA) differ from those in the Western world. These unique systems developed by Chinese doctors may inspire the refining of global clinical practice. We reviewed the efficacy of allo-HSCT practice from available Chinese studies on behalf of the HSCT workgroup of the Chinese Society of Hematology, Chinese Medical Association and compared these studies to the consensus or guideline outside China. We summarized the consensus on routine practices of all-HSCT in China and focused on the recommendations of indications, conditioning regimen, and donor selection.

## Background

Allogeneic stem cell transplantation (allo-HSCT) is widely used to treat malignant hematological neoplasms and non-malignant hematological disorders [1–4]. The Chinese Blood and Marrow Transplantation Registry (CBMTR) reported that the total number of allo-HSCT cases increased steadily from 950 cases in 2008 to over 5000 cases in 2016 [5, 6]. In contrast, 6189 allo-HSCT cases were performed in Europe and 8351 cases were performed in the USA in 2015 [3, 7]. Therefore, the standardization of allo-HSCT practices in China would provide a major global impact based on the large patient population.

The significant growth of allo-HSCT is a result of the increased availability of alternative donors and refinement of indications. First, there was a shortage of human leukocyte antigen (HLA)-matched sibling donors and unrelated donors in China, but the success of haploidentical HSCT (HID-HSCT) ushered in a new era of “everyone has a donor” [8]. A total of 99% of HID-HSCT cases followed the “Beijing Protocol”, which includes T-replete myeloablative HID-HSCT with granulocyte colony-stimulating factor (G-CSF) and anti-thymocyte globulin (ATG) [9–23]. The Baltimore group introduced HID-HSCT followed by the posttransplant cyclophosphamide (PT-CY) regimen, which was also implemented in China [24]. The number of HID-HSCT cases increased to approximately 2500 annually in 2016, which made it the largest source of allo-HSCT donors (37.6–51.5%) in China since 2013 [5, 8, 25] and take 40% of annual HID-HSCT cases worldwide. In contrast, the frequency of HID-HSCT grew steadily from 3 to 5% to approximately 10% of allo-HSCT in Europe (2000 HID-HSCT cases in 2015) and the USA (1000 HID-HSCT cases in 2015) [3], where HID remains a minor donor source compared to HLA-matched sibling donor (MSD) and matched unrelated donors (MUD). The rapid development of HID-HSCT also raised questions of “Who is the best alternative donor?” and even “Who is the best donor?” because HLA no longer plays the predominant role in donor selection [10, 16, 26–28]. Second, individualized conditioning regimens based on patients’ statuses expand the target patient population, such as a reduced-intensity regimen for older patients [29–31] or novel conditioning for severe aplastic anemia (SAA) [32–34]. Third, pre-transplant risk stratification has enabled the early identification of patients with high risk of relapse in chemotherapy, which may provide valuable information for the selection of allo-HSCT as post-remission therapy [35, 36].

The Chinese Society of Hematology (CSH) updated the recommendations from a consensus conference of the HSCT workgroup of CSH in 2017 based on differences in the practices of allo-HSCT in China and the Western world. The present guidelines focus on indications, conditioning regimen, and donor selection. Seventeen experts with recognized clinical and research expertise in allo-HSCT participated in the consensus discussion and were elected as members of the HSCT workgroup, Chinese Society of Hematology. These experts represented the most active allo-HSCT centers (approximately 60% of total allo-HSCT cases) in China. This consensus will likely contribute to the standardization of allo-HSCT practices in China and become an inspiration for further international cooperation to refine global practices.

### Indication and timing of allo-HSCT

Other HSCT groups, such as the American Society for Blood and Marrow Transplantation (ASBMT) and British Society of Blood and Marrow Transplantation, have systemically summarized the indications and timing for allo-HSCT or recommended guidelines for specific diseases, such as the National Comprehensive Cancer Network (NCCN) guidelines for malignant hematological diseases and the British Committee for Standards in Haematology (BCSH) guidelines for aplastic anemia. Recent Chinese studies suggested that specific patient subgroups may benefit from allo-HSCT rather than other conventional non-allo-HSCT treatments. Therefore, the indications for allo-HSCT may be extended for these patient subgroups in China, which is not in strict accordance with the current recommendations in the Western world (Table 1).

**Table 1:** The clinical outcome of HID-HSCT and trials comparing allo-HSCT with chemotherapy or TKI

Author [ref.]	Patients	No	Diagnosis	CIR	OS	LFS
Wang et al. [27], 2013	HID-HSCT, retrospective	756	Leukemia	15% (SR), 26% (HR) at 2 years		68% (SR) at 3 years 49% (HR) at 3 years
Huang et al. [15], 2012	HID-HSCT vs CT prospective	58 vs 74	AML-CR1 adults IR or HR	12 vs 57%*	7.5 vs 54.7%* at 4 years	73.1 vs 44.2%* at 4 years
Zhu et al. [35], 2013	HSCT vs CT	58 vs 58	ETO(+) adults AML-CR1	22.1 vs 78.9% (HR)* 14.7 vs 5.3% (LR)	71.6 vs 26.7% (HR)* 75.7 vs 100% (LR)	61.7 vs 19.6% (HR)* 70.3 vs 94.7% (LR)
Qin et al. [36], 2015	HSCT vs CT	57 vs 29	In(16) adults AML-CR1	7.1 vs. 87.7% (poor MR)*; 0 vs. 26.9% (good MR);	93.3 vs. 40.0%, (poor MR)*; 72.9 vs. 77.1% (good MR)	86.7 vs. 12.3% (poor MR)* 72.9 vs. 73.1% (good MR)
Yan et al. [82], 2014	HID-HSCT vs CT	79 vs 59	SR-ALL-CR1 adults	29.9 vs 66.3%* at 5 years	70.4 vs 28%* at 5 years	54.4 vs 23.9%* at 5 years
Sun et al. [83], 2014	HID-HSCT vs CT	79 vs 104	HR-ALL-CR1 adults	18.7 vs 60.5%* at 3 years	72.5 vs 26.6%*; at 3 years	63.9 vs 21.1%* at 3 years
Wang et al. [84], 2012	HID-HSCT, T-ALL	72	T-ALL	18.8% (CR1) 37.5% (non-CR1)		54.8% (CR1) 12.5% (non-CR1)
Xu et al. [46], 2016	HSCT	52	Pediatric T-ALL(HR)	32.7% at 3 years	55.5% at 3 years	54.1% at 3 years
Jiang et al. [85], 2011	HSCT vs IM	87 vs 45	CML-AP adults		81.2 vs 100% (LR) at 6 years 81.3 vs 61.3% (IR)* at 6 years 100 vs 17.7% (HR)* at 5years	80.7 vs 80.9% (LR) at 6 years, 61.9 vs 47.1% (IR)* at 6 years 66.7 vs 9.3% (HR)* at 5 years
Xu et al. [52], 2016	HSCT vs TKI2	60 vs 33	CML-AP adults		86.4 vs. 42.9%* at 5 years	76.1 vs. 14.3%* at 5 years

*HID* haploidentical donor, *MRD* matched-related donor, *HSCT* hematopoietic stem cell transplantation CT, *AML* acute myeloid leukemia, *ALL* acute lymphoblastic leukemia, *MDS* myelodysplastic syndrome, *LR* low risk, *IR* intermediate risk, *HR* high risk, *T_ALL* T cell ALL, *MR* molecular response, *ph* Philadephia chromosome[t(9;22)], *CML* chronic myeloid leukemia, *CP* chronic phase, *AP* accelerated phase, *BC* blast crisis, *IM* imatinib, *TKI2* second-generation TKI, *SAA* severe aplastic anemia. * The difference was significance between two groups (*P*<0,05)

#### Acute myeloid leukemia

Allo-HSCT, especially MSD-HSCT, is the standard care option for acute myeloid leukemia (AML) patients classified as an intermediate and high-risk karyotype by the NCCN stratification system [37] in any disease state (CR1/CR2 or above/active disease) in the ASBMT recommendations [38]. HID-HSCT was confirmed as an equally good option to MSD-HSCT as post-remission therapy for AML patients in CR1 who lack a matching donor following the Beijing Protocol [18]. A prospective, multicenter study investigated 450 AML patients who were assigned to undergo haplo (231 patients) or ISD HSCT (219 patients) based on donor availability. The HID and ISD HSCT groups exhibited, respectively, comparable 3-year disease-free survival (DFS) of 74 and 78% (*p* = 0.34), overall survival (OS) of 79 and 82% (*p* = 0.36), cumulative incidence of relapse (CIR) of 15 and 15% (*p* = 0.98), and treatment-related mortality (TRM) of 13 and 8% (*p* = 0.13). Therefore, we did not differentiate recommendations for transplant indications based on donor source (i.e., related donor, unrelated donor, umbilical cord blood, or haploidentical donor) (Table 2).

**Table 2:** The clinical results of HID and HID comparing with MRD or URD or CBT

Author	HSCT type	Case number	Diagnosis	TRM	CIR	aGVHD	OS	LFS
Wang et al. [18], 2015	HID vs MSD multicenter, prospective	231 vs219	Adults IR/HR AML-CR1	3 years, 13 vs 8%	3 years, 15 vs 15%		3 years, 79 vs 82%	3 years, 74 vs 78%
Liu et al. [61], 2013	HID vs MSD	212 vs 46	Pediatric AL			II–IV* 40.8 vs 20%; III–IV 14.3 vs 16.9%	77.8 vs 65.5%	68.9 vs 52.5% (ALL-CR1); 82.5% vs 71.7% (AML-CR1)
Wang et al. [17], 2016	HID ns MSD Adults Phase II randomizes	121 vs 89	(Ph-)ALL-CR1(HR)		3 years, 18 vs 24%			3 years, 61 vs 60%
Chen et al. [43], 2015	HID vs MSD	101 vs 38	Ph + ALL	15.6% at 5 years	20.3% at 5 years		74.0 vs 68% at 5 years	65.8 vs 61% at 5 years
Gao et al. [44], 2015	HID vs MSD	47 vs 35	Ph + ALL	21.3 vs 17.1%	19.1* vs 44.8%.	III–IV 17.0 vs 11.4%	2 years, 63.8 vs 62.6%	2 years, 59.5 vs 45.7%
Wang et al. [19], 2016 CBMTR data based	HIDs vs MRD	136 (3/6) vs 90(4/6) vs 228	MDS				58 vs 63 vs 73%	58%*, 63 vs 71%
Ma et al. [64], 2016	HID vs MSD	67 vs 23	CML-BC				3 years, 60.0 vs 55.3%	3 years, 51.1 vs 47.8%
Xu et al. [33], 2016	HID vs MSD\prospective	101 vs 48	SAA failure to previous IST			II–IV* 33.7 vs 4.2%; III–IV, 7.9 vs 2.1%	3 years, 89.0 vs. 91.0%	3-year FFS 86.8 vs. 80.3%
Xu et al. [34], 2017	HID vs MSD Data based	89 vs 69	SAA, upfront	97.8 vs 97.1%		II–IV* 30.3 vs. 1.5%, III–IV* 10.1 vs. 1.5%	3 years, 86.1 vs 91.3%	3-year FFS 85.0 vs 89.8%
Sun [79], 2016	HID vs URD, Pair-match	87 vs 87	AML-CR1	13.8 vs 15.7%	12.7 vs 24%	III–IV 9.2 vs 9.4%	5 years, 78.2 vs 63.6%	5 years, 73.5 vs 60.3%
Huang et al. [13], 2009	HID vs URD	219 vs 78	Malignant	2 years, 20 vs 18%	2 years, 12 vs 18%	II–IV 47 vs 31%	4 years, 74 vs 74%	4 years, 67 vs 61%
Han et al. [39], 2017	HID, vs MSD vs MUD	127 vs 144 vs 77	Adult patients	16.4 vs 11.6 vs 19.6%	14.8 vs 21.1 vs 16.7%	III–IV 11.4 vs 7.7 vs 13.5%	70.1 vs, 73.7 vs 69.8%	5-years DFS 68.7 vs 67.3 vs 63.7%
Luo et al. [23], 2014	HRD vs URD vs MSD prospective ChiCTR-OCH-12002490	99 vs 116 vs 90	Hematologic malignancies	30.5 vs 22 vs 4.7%*	14.2* vs 21.2 vs 34%; 15.4* vs 28.2 vs 49.9% (in HR pts)	II–IV 42.4 vs 39.7 vs 15.6%	60.8 vs 63.5 vs 77.2%,	58.3 vs 58.4, vs 63.6%
Mo et al. [81], 2016	HID vs CBT multiple centers	65 vs 64	ALL (HR) pediatric	2 years, 12.8 vs 18.8%	2 years, 16.1 vs 24.1%		2 years, 82 vs 69.6%	2 years, 71 vs 57.2%*

*OS* overall survival, *RR* relapse rate, *LFS* leukemia-free survival, *HID* haploidentical donor, *MRD* matched related donor, *HSCT* hematopoietic stem cell transplantation, *MRD* matched related donor, *CBT* cord blood transplantation, *URD* unrelated donor, *AML* acute myeloid leukemia, *ALL* acute lymphoblastic leukemia, *MDS* myelodysplastic syndrome, *LR* low risk, *IR* intermediate risk, *HR* high risk, *T_ALL* T cell ALL, *MR* molecular response, *ph* Philadephia chromosome[t(9;22)], *CML* chronic myeloid leukemia, *CP* chronic phase, *AP* accelerated phase, *BC* blast crisis, *IM* imatinib, *TKI2* second-generation TKI, *SAA* severe aplastic anemia. * The difference was significance between two groups (*P*<0.05)

AML patients classified as a low-risk karyotype by the NCCN guidelines, such as patients with genetic abnormality of RUNX1-RUNX1T1 and CBFB-MYH1, may benefit from allo-HSCT in CR1 by risk-directed, minimal residual disease (MRD)-based therapy. An AML05 multicenter trial revealed that MRD status (RUNX1-RUNX1 reduction < 3 log units) after the second consolidation discriminated these patients into subgroups. The high-risk group was defined as patients in whom major molecular remission (MMR) was not achieved after the second consolidation therapy or patients who exhibited loss of MMR within 6 months of achieving it. The low-risk subgroup was defined as patients in whom MMR was achieved after the second consolidation therapy and maintained for 6 months. Allo-HSCT reduced relapse and improved survival compared to chemotherapy in these high-risk patients (HSCT vs. chemotherapy: respectively, CIR 22.1 vs 78.9%, *P* < 0.0001; DFS 61.7 vs. 19.6%, *p* = 0.001), whereas chemotherapy/auto-HSCT achieved a low relapse rate (5.3%) and high DFS (94.7%) in low-risk patients. MRD-directed pre-transplant risk stratification may improve the outcome of t(8;21) AML in CR1 [35]. Similar results were observed with inv(16) AML. Poor molecular response was defined as a CBFB-MYH11 level of 0.2% after the second consolidation. Allo-HSCT decreased the 3-year CIR and increased the DFS and OS of patients who exhibited a poor molecular response [36]. Therefore, patients with t(8;21) or inv(16) AML, who are considered high-risk by MRD-directed risk stratification, benefited from HSCT in CR1.

The detection of some molecular markers, such as semi-quantitative assessment of FLT3-ITD allelic ratio, was not widely available in China, and the risk stratification generally followed NCCN rather than European Leukemia Net (ELN) recommendations.

#### Acute lymphoblastic leukemia

Adult patients with high Ph(−) Acute lymphoblastic leukemia (ALL) in CR1 benefit from MSD-HSCT and HID-HSCT (Table 2). Han et al. retrospectively investigated the outcomes of HID-HSCT in adults with standard-risk ALL in CR1 and compared these patients to MSD and MUD patients. A total of 127 HID, 144 MSD, and 77 MUD recipients were included in the study. There were no differences in grade III–IV acute graft-versus-host disease (aGVHD) (11.4 vs. 7.7 vs. 13.5%, *p* = 0.468), 5-year TRM (16.4 vs. 11.6 vs. 19.6%, *p* = 0.162), 5-year CIR (14.8 vs. 21.1 vs. 16.7%, *p* = 0.231), 5-year OS (70.1 vs. 73.7 vs. 69.8%, *p* = 0.525), 5-year DFS (68.7 vs. 67.3 vs. 63.7%, *p* = 0.606), or 3-year GVHD-relapse-free survival (GRFS; 50.8 vs. 54.9 vs. 52.2%, *p* = 0.847), respectively, [39]. Wang et al. compared HID and MSD for HSCT in adults with Ph(−) high-risk ALL in a biological phase III randomized multicenter study [40]. A total of 103 cases received HSCT from HID and 83 received HSCT from MSD. There were no differences in 3-year DFS (61 vs. 60%, *p* = 0.91) from CR, 3-year OS (68 vs. 64%, *p* = 0.56) from HSCT, TRM (13 vs. 11%, *p* = 0.84), or CIR (18 vs. 24%, *p* = 0.30). Therefore, HID-HSCT is a valid alternative as post-remission treatment for high- and standard-risk adult patients with ALL in CR1 who lack an identical donor [17].

Ph + ALL remains an important indication for allo-HSCT in this era of treatment with tyrosine kinase inhibitors (TKIs) [40, 41]. The results of MSD and HID-HSCTs were similar in adult and pediatric patients [42, 43]. Chen et al. investigated 50 pediatric patients with Ph + ALL who underwent HID-HSCT. The 5-year EFS was 61.0%, the OS was 70.0%, the 3-year CIR was 22.7%, and the NRM was 16.4%. Therefore, HID-HSCT for pediatric patients with Ph + ALL yielded promising long-term survival [42]. Zhang et al. analyzed the outcomes of 82 Ph + ALL patients who underwent HID-HSCT (*n* = 47) or MSD-HSCT (*n* = 35). HID-HSCT was associated with a significantly lower relapse rate than MSD-HSCT (44.8 vs. 19.1%, *p* < 0.05). There were no differences in NRM, LFS, or OS between the two groups [44]. Recently, Wang et al. reported for low-risk Ph + ALL patients, who were defined as WBC < 30 × 109/L at diagnosis and 3-log reduction of BCR-ABL levels from baseline after two consolidation cycles, there was no significant difference between the allo-HSCT and non-transplant groups for CIR (8.5 vs. 7.7%, *p* = 0.671), DFS (88.2 vs. 83.9%, *p* = 0.426), and OS (96.6 vs. 83.3%, *p* = 0.128), which suggested selected low-risk patients might be free from allo-HSCT [45].

Pediatric patients with T-ALL benefit from HSCT, including HID-HSCT. Xu ZL examined 48 consecutive children with high-risk T-ALL who underwent HID-SCT in a prospective study [46]. The 3-year CIR was 30.8%, the NRM was 14.7%, and the 3-year LFS was 54.4%. Children who received transplants during CR1 exhibited a higher LFS (65.7 vs. 26.0%, *p* = 0.008) and lower relapse rate (19.8 vs. 56.7%, *p* = 0.014) than children who received transplantation during non-CR1. Therefore, HID transplantation is a valid alternative in standard- and high-risk adults with ALL in CR1 who lack matched donors.

The rapid progression of immunotherapies, including cellular therapies such as CAR-T, supports the importance of the incorporation of these immunotherapies with allo-HSCT, especially for refractory ALL, before HSCT or MRD+ ALL post-HSCT [47].

#### Myelodysplastic syndromes (MDS)

Allo-HSCT is the standard care for advanced MDS (IPSS Intermediate-2/high-risk). Patients with lower-risk MDS (refractory anemia or refractory anemia with ringed sideroblasts) who exhibited poor prognostic features and/or signs of progression or sustained profound cytopenia (neutrophil count < 0.5 × 10^9^/L and/or platelet count < 20 × 10^9^/L) were also considered candidates for allo-HSCT.

Wang et al. analyzed the outcomes of 454 patients with MDS who underwent HSCT from HIDs (*n* = 226) or ISDs (*n* = 228) and reported the results to the CBMTR. The 4-year NRM values of the 3/6 HID (*n* = 136), 4–5/6 HID (*n* = 90), and ISD patient groups were, respectively, 34, 29, and 16% (*p* = 0.004); the CIR values were 6, 7, and 10% (*p* = 0.36); the 4-year OS values were 58, 63, and 73%, (*p* = 0.07); and the RFS values were 58, 63, and 71% (*p* = 0.14). HLA disparity exerted no effect on survival in the HID group [19].

#### Chronic myelogenous leukemia

Allo-HSCT is no longer the primary treatment option for chronic myelogenous leukemia (CML) patients in the early chronic phase because imatinib exhibited better outcomes than MSD-HSCT in young persons with newly diagnosed CML-CP [48]. The corresponding percentage of allo-HSCT for CML patients decreased from 22% in 2008 to less than 2% in 2017 in China. However, allo-HSCT was superior to first- and second-generation tyrosine kinase inhibitors (TKIs) for patients in the accelerated phase (AP) and blastic crisis (BC) [49–51]. As anti-T315i-mutated TKIs are not available in China, and allo-HSCT is an option for this group of patients in any phase. Xu et al. reported the outcome of SCT in 22 patients with T315I(+) CML, and most of whom (*n* = 16) underwent HID-SCT. The 2-year LFS were 80.0, 72.9, and 0% for the CP, AP/AP-CPn, and BP/BP-CPn groups, respectively, at the time of SCT [52].

#### Severe aplastic anemia

The Guidelines of the British Society for Hematology list MSD-HSCT and MUD-HSCT as first-line standard care for severe aplastic anemia (SAA) patients (age < 50), and HID-HSCT was only considered a second-line treatment for refractory SAA after a failed immunosuppressant therapy (IST). In contrast, transplant indications for SAA are not differentiated on donor source in China. The feasibility of HID transplantation for the treatment of SAA patients after the failed immunosuppressant therapy was evaluated in a prospective multicenter clinical trial [33] (Table 2). Recipients of HID-HSCT exhibited a higher incidence of grade II–IV aGVHD than patients (*n* = 48) who received MSD-HSCT (33.7 vs. 4.2%, *p* < 0·001) but similar values of grade III–IV aGVHD (7.9 vs. 2.1%, *p* = 0.157), 3-year OS (89.0 vs. 91.0%, *p* = 0.555), and failure-free survival (FFS) (86.8 vs. 80.3%, *p* = 0.659). Furthermore, HID-HSCT was evaluated as an upfront therapy for SAA in a registry-based comparison study. Eighty-nine patients received HID-HSCT, and 69 patients received MSD-HSCT. HID recipients exhibited a similar incidences of extensive cGVHD (3.4 vs. 0%, *p* = 0.426), 3-year OS (86.1 vs. 91.3%, *p* = 0.358), and 3-year FFS (85.0% vs. 89.8%, *p* = 0.413) compared to MSD, with increased incidence of II-IV aGVHD (30.3% vs. 1.5%, *P* < 0.001) and total cGVHD (30.6 vs. 4.4%, *p* < 0.001). Xu et al. reported that the treatment of 52 children with SAA with HID-HSCT produced 3-year OS of 84.5 and FFS of 82.7% [53]. These results suggest that newly diagnosed and refractory SAA benefit from HID-HSCT as MSD or MUD-HSCT.

In summary, the published evidence suggests that allo-HSCT be recommended for intermediate- and high-risk AML-CR1, selected subgroups of low-risk AML-CR1, Ph + ALL, adult standard-risk ALL- CR1, MDS, high-risk AL, and CML-AP/CBL-BC. HID-HSCT and/or MUD-HSCT demonstrated equivalent outcomes as MSD-HSCT in China. Therefore, we did not differentiate recommendations for transplant indications based on donor source, which is different from previous recommendations from Western countries.

Systematic, standardized pre-transplant risk stratification is important for patients who are eligible for allo-HSCT. The European Group for Blood and Marrow Transplantation (EBMT) risk score and hematopoietic cell transplantation-specific comorbidity index (HCT-CI) were feasible for the predicting of patient outcomes following HID-HSCT in China [54, 55]. A modified EBMT risk score that used the number of HLA disparity instead of donor type also predicted patient outcomes [56].

Select older patients (age > 50) with low HCT-CI (<= 2) and good performance status tolerated myeloablative haplo-HSCT with similar outcomes as younger adults [57]. Haplo-HSCT with a reduced-intensity regimen (RIC) with substitution of cyclophosphamide with fludarabine (Flu) was feasible in patients above 60 years of age, who exhibited similar engraftment and relapse rates as myeloablative conditioning in China [29, 30]. Select adults ≥ 70 years with hematological malignancies are considered for transplant in the USA. [31]. Therefore, the present consensus does not provide specific recommendation for eligible age.

### Recommendation: indications and timing for allo-HSCT

#### Patients with non-malignant hematological diseases

1. AML include non-APL AML and APL.
2. AML (non-acute promyelocyte leukemia (APL)
  A. AML (non-APL in CR1):
    - Intermediate- or unfavorable-risk disease according to NCCN risk stratification.
    - Patients who achieve CR1 after ≥ 2 cycles of therapy.
    - Patients with AML showing myelodysplasia-related changes or therapy-related myeloid changes.
    - Patients with favorable-risk diseases according to WHO risk stratification who exhibit a poor molecular response to chemotherapy. RUNX1-RUNX1 reduction < 3 log units or CBFB-MYH11 level ≥ 0.2% after two consolidation cycles.
  B. AML (non-APL) ≥ CR2
  C. AML (non-APL) not in remission: allo-HSCT as salvage therapy with individualized conditioning regimens.
3. APL:
  A. Patient fails to react to induction therapy.
  B. Relapsed APL patient (molecular, cytogenetic, or hematological relapse) who remains PML-RARA-positive after the second induction therapy.
4. Acute lymphocytic leukemia (ALL)
5. Acute lymphocytic leukemia (ALL) included ≦ 14 and > 14 years old patients [40, 58–60].
  A. ALL-CR1: especially patients with MRD(+) 8 weeks after induction therapy or showing high-risk factors: age > 35; high WBC count at presentation (≥ 100 × 10^9^/L for T lineage and ≥ 30 × 10^9^/L for B lineage); t(9;22) or complicated chromosome.
  B. The decision for allo-HSCT for adolescents who receive a chemotherapy protocol for pediatric patients should be made based on the appropriate guidelines for ALL (age ≤ 14 years).
  C. ALL ≥ CR2
  D. ALL not in remission: allo-HSCT as salvage therapy with individualized conditioning regimens or refer to clinical trials with novel cellular therapies as a bridge with allo-HSCT.
6. Acute lymphocytic leukemia (ALL) age ≤ 14 years [40, 61].
  A. Adolescent and pediatric ALL in CR1
    - Patients who fail to achieve hematological CR or MRD > 1% within 28–30 days.
    - Patients achieve CR with MRD > 0.1% within 12 weeks after therapy.
    - Patients who exhibit MLL rearrangements who are < 6 months old or with WBC count > 300 × 10^9^ cells/L.
    - Philadelphia chromosome-positive patients, especially patients exhibiting poor response to prednisone and positive MRD at any time 4–12 weeks after therapy.
  B. Allo-HSCT in ALL ≥ CR2: All patients who exhibit very early or early relapse are candidates for allo-HSCT during CR2. All patients in CR3 are also recommended for allo-HSCT.
  C. ALL not in remission: allo-HSCT as salvage therapy for refractory or relapsed ALL.
7. Chronic myeloid leukemia (CML) [48, 62–64]:
  A. Allo-HSCT could be considered if patients in CP phase fail to respond to TKIs, depending on their age, consent, and the will of the patient.
  B. Patients who are resistant to or intolerant of second-generation TKIs are recommended to receive allo-HSCT.
  C. Patients with T315I-mutated BCR-ABL should choose allo-HSCT as a first-line therapy.
  D. Patients who have progressed to the accelerated or blast crisis phase are recommended for allo-HSCT.
8. Myeloproliferative neoplasms, including MDS, MDS/MPN, CMML, atypical CML, JMML, MDS/MPN, or unclassified [19, 65].
  A. Patients with intermediate-2 or high-risk IPSS scores are recommended for early allo-HSCT [66].
  B. Patients with low-risk or intermediate-1 IPSS scores but showing severe neutropenia or thrombopenia or patients who are transfusion-dependent.
  C. Children with JMML.
9. Myelofibrosis (MF): Patients with primary or secondary myelofibrosis with intermediate-2 or high-risk scores are candidates for allo-HSCT. The IPSS and DIPSS scores refer to NCCN guidelines [65].
10. Multiple myeloma (MM): [67]
  A. Allo-HSCT is recommended for young patients with high-risk cytogenetic changes, such as t(4;14);t(14;16);17p-. Patients exhibiting disease progression after initial auto-HSCT may also receive allo-HSCT as salvage therapy.
11. Hodgkin lymphoma (HL): Patients who are refractory or relapse after ≥1 course of auto-HSCT [68].
12. Non-Hodgkin lymphoma (NHL): [69]
  A. Chronic lymphocytic leukemia/small lymphocytic lymphoma (CLL/SLL): Allo-HSCT should be considered for young patients under the following conditions, in the absence of newly available drugs.
    - Patients who are refractory to purine analogs or relapse within 12 months.
    - Patients who respond to auto-HSCT or purine analog-containing regimens but relapse within 24 months.
    - Patients with high-risk cytogenetic or molecular factors, regardless of response to therapy or relapse.
    - Patients exhibiting symptoms of Richter syndrome.

Others: Allo-HSCT is also recommended for patients with NHL, including follicular lymphoma, diffuse large B-cell lymphoma (DLBCL), mantle cell lymphoma, lymphoblastic cell lymphoma and Burkitt lymphoma, peripheral T-cell lymphoma, and NK/T-cell lymphoma who are refractory, relapsed, or in ≥ CR2. If HLA-matched donors are available, HSCT may also be considered in CR1 for adult patients with mantle cell lymphoma, lymphoblastic cell lymphoma, Burkitt lymphoma, peripheral T-cell lymphoma, and NK/T-cell lymphoma.

#### Patients with non-malignant hematological diseases

1. Aplastic anemia (AA): [33, 34, 70]
  A. Newly diagnosed with severe aplastic anemia (SAA): Patients who are < 50 years of age with SAA or vSAA with HLA-matched sibling donors could receive allo-HSCT as first-line therapy. Pediatric SAA/vSAA patients with ≥9/10 loci-matched unrelated donors may also choose allo-HSCT as first-line therapy. HID HSCT is recommended for young patients without MSD.
  B. Refractory and/or relapsed SAA:
    1. SAA or vSAA patients below the age of 50 years who fail to respond to immunosuppression therapy (IST) or relapse may choose to undergo HID, MUD, or CBT
    2. SAA or vSAA patients who are 50–60 years old and fail to respond to IST or relapse with ECOG scores ≤2 are recommended for MSD or MUD transplantation.
  C. Transfusion-dependent non-SAA patients, based on the guidelines for SAA.
2. Paroxysmal nocturnal hemoglobinuria (PNH): SAA/PNH who failed in IST.
3. Thalassemia: Allo-HSCT is recommended for transfusion-dependent severe thalassemia, including severe thalassemia, hemoglobulin E combined with thalassemia, and severe hemoglobulin E disease. HSCT is recommended before progression to stage 3 for children (2–6 years old).
4. Fanconi anemia: HSCT should be performed before the disease progresses to MDS or leukemia and too many blood transfusions.
5. Others: Patients with congenital immune deficiencies or metabolic diseases, including severe combined immunodeficiency and mucopolysaccharidoses, should receive HSCT.

All patients eligible for allo-HSCT should be evaluated using the HCT-CI, Kanofsky or Lansky Play performance score, EBMT score, or modified EBMT score for Haplo-EBMT.

### Donor selection and graft source

**Fig. 1:**
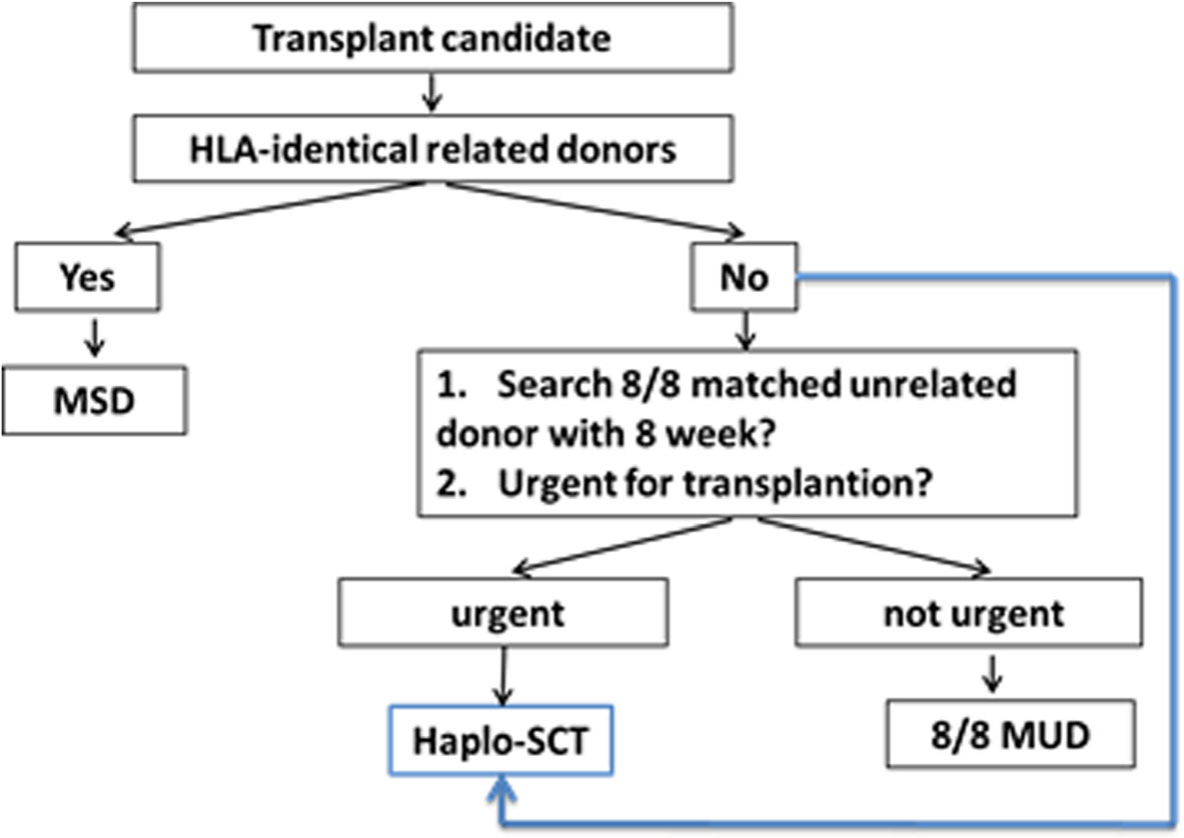
Donor research flowchart for allo-HSCT

MSD are generally the preferred choice for allo-HSCT, and Haplo, MUD, and cord blood (CB) are alternatives. The ideal donor should be identified among alternative donor candidates based on factors such as recipient condition (refractory or relapsed status, age, and performance status), characteristics of the alternative donors, and the experience of the transplantation center (if HID-HSCT available) (Fig. 1).

#### HID-HSCT

HID-HSCT exhibits a similar clinical outcome as MSD and MUD in the treatment of AML, ALL, MDS, and SAA, which are supported by the multicenter prospective studies listed in Table 2. The characteristics of haplo donor: (1) almost all patients can find a haplo donor on time and often have more than one alternative candidate, (2) a haplo donor is more suitable for patients in need of urgent allo-HSCT because it is not time-consuming, with only 2–3 weeks required for HLA-typing and physical examination, (3) HID can donate enough graft cells to be stored for future cell therapy, especially for high-risk relapsed patients, (4) bone marrow and peripheral stem cells may be obtained based on the clinical condition, (5) HID-HSCT exhibits a lower incidence of relapse than MSD-HSCT or MUD-HSCT in high-risk malignant hematological patients, (6) the incidence of aGVHD is higher in HID-HSCT than MSD-HSCT, and (7) peripheral blood and/or bone marrow are feasible in HID-HSCT.

Wang et al. analyzed the outcomes of 1210 consecutive transplant cases treated with a uniform protocol and proposed an algorithm for the selection of an HID from more than one candidate. Younger donors and male donors were associated with low NRM (HR = 0.30, *p* = 0.008 and HR = 0.65, *p* = 0.002) and high OS (HR = 0.73, *p* = 0.033 and HR = 0.73, *p* = 0.005). Father donors were associated with low NRM (HR = 0.65, *p* = 0.02) and aGVHD (HR = 0.69, *p* = 0.001) and high OS (HR = 0.66, *p* = 0.003) compared to mother donors. Child donors were associated with lower aGVHD than sibling donors (HR = 0.57, *p* = 0.01). Older sister donors were inferior to father donors for NRM (HR = 1.87, *p* = 0.02) and OS (HR = 1.59, *p* = 0.03). Non-inherited maternal antigen (NIMA)-mismatched sibling donors were associated with the lowest incidence of aGVHD compared with parental and non-inherited paternal antigen (NIPA)-mismatched sibling donors. Specific HLA disparities were not significantly correlated with the outcomes. The order rank for haplo donor characteristics in this algorithm was young, male, and non-inherited maternal antigen mismatch. Transplants from older mothers and NIPA-mismatched donors should be avoided as much as possible [28, 71].

Chang et al. focused on the relationship between donor-specific anti-HLA antibodies (DSAs) and primary graft failure (GF) after HID-HSCT and designed a prospective study with randomly assigned training and validation sets. The incidence of primary GF was 6.4%, which included GR (0.9%) and PGF (5.5%). Multivariate models revealed that DSAs (median fluorescence intensity (MFI) ≥ 10,000) correlated to primary GR (*p* < 0.001), and DSAs (MFI ≥ 2000) strongly associated with primary PGF (*p* = 0.005). These results suggested that the incorporation of DSAs into the algorithm improved HID selection. Donors with DSA MFI > 10,000 should be avoided [71, 72].

Donor-recipient CMV serostatus matching was not associated with transplant outcomes in HID-HSCT following the Beijing protocol. In contrast, the Baltimore group suggested that donors should have aCMV IgG serologic status similar to that of the recipients. This discrepancy may be related to the higher incidence of CMV infections in Chinese compared to Western populations.

Cohort studies that considered the different models of NK-KIR alloreactivity (KIR ligand model, missing-ligand model, KIR-KIR model, and receptor-ligand model) reported discordant results between different HID-HSCT approaches. A high relapse rate was associated with missing self-molecules or missing ligands in the Beijing protocol, and a benefit of using donors with KIR B haplotypes was found in the PT-CY protocol. Both of these protocols are different from the “Perfect mismatch” in T-cell-depleted HID-HSCT [73–76].

The modified G-CSF + ATG protocols with pure G-BM or G-PB were also feasible compared to the mixed grafts of G-PB and G-BM in HID-HSCT introduced by the Beijing group [21, 77]. A propensity score method-based multicenter study demonstrated that HID-HSCT with mixed grafts achieved better 3-year DFS compared to G-PB alone (59.9 vs. 44.3%) [78].

#### MUD-HSCT

MUD-HSCT exhibits similar clinical outcomes as MSD-HSCT and HID-HSCT [79]. However, there are several specific characteristics in China for MUD: (1) the probability of finding an appropriate donor is very low (approximately 11% compared to 40–70% in the Western world) [80], and bone marrow graft is not available for MUD in China, (2) searching for and preparing a MUD would require 3–6 months, (3) donors may opt out of donation at any time, (4) there is very little chance that the donor would be willing to re-donate lymphocytes or stem cells if the patient needs it, and (5) there is a decreased incidence of severe aGVHD and increased risk of relapse in MUD transplantations compared to HID transplantations.

#### CB-HSCT

The treatment outcome is similar to MUD transplantation for malignant hematological diseases [81]. The following characteristics of CBT are noted: (1) it is possible to search and prepare CB on time without delays, (2) the incidence and severity of GVHD is low, (3) blood reconstruction is generally delayed, which leads to a high risk of infection, and (4) single-dose CBT is generally used for child patients, and double-dose CBT has not been widely adopted in China.

Who is the best allo-donor for a patient with acute leukemia for transplantation in CR1? Wang et al. reported a prospective data set of 1199 consecutive subjects who received a transplant from HID (*n* = 685) or MSD (*n* = 514). The 3-year LFS values were similar (75 vs. 74%, *p* = 0.95). Three major risk factors for TRM were identified in the multivariate model: higher donor/recipient age ratio, female-to-male transplants, and donor-recipient ABO major-mismatch transplants. Therefore, the donor-recipient age, matching for gender, and ABO incompatibility should be considered before selecting the ideal donor for patients with acute leukemia receiving related transplants in our model [16].

HLA-matched sibling donors are generally the preferred choice for allo-HSCT. However, we found that HID may be the ideal donor for subgroups with high risk or MRD-positive patients because HID-HSCT exhibited a strong graft versus leukemia (GVL) effect. MSD-HSCT is generally considered the best allo-HSCT. Chang et al. performed a retrospective study (*n* = 339) and a prospective study (*n* = 340) to verify this relationship [82]. MRD was determined using multiparameter flow cytometry. The results indicated that haplo-SCT was associated with a lower incidence of relapse and better survival for pre-MRD-positive AML patients, which suggests a stronger anti-leukemia (GVL) effect than MSD-HSCT.

### Recommendations: donor selection and mobilization

#### General principle of donor selection

HLA-matched sibling donors are the first choice as allo-HSCT donors. A related HID is required for patients at high risk of relapse or search for MUD when an MSD is not available. Patients with favorable-risk diseases may choose unrelated donors because post-transplant cellullar therapy is not necessary. Child patients may also choose CBT.

Several factors, including conditioning regimens, donors, disease status, and performance status of the patient, affect the outcome of the transplantation. Clinical practices must be standardized, and management strategies for each patient must be individualized. The optimal procedure includes risk stratification at diagnosis, overall treatment schedule, and transplantation at the optimal times.

#### Algorithm for HID

Haploidentical donors may be chosen in this order: children, male sibling, and father, mismatched sibling with non-inherited maternal antigen (NIMA), mismatched sibling with non-inherited paternal antigen (NIPA), mother, and other collateral relatives. Donors with donor-specific anti-HLA antibodies (DSAs) median fluorescence intensity (MFI) > 10,000 should also be avoided. ABO and CMV IgG serological status compatibility between donor and recipient are preferred. A KIR ligand match is preferred in HID-SCT following the Beijing protocol.

#### Algorithm for MUD

MUD requires HLA matching in high resolution. Nine to 10 loci matches are needed for HLA-A, B, C, DRB1, and DQ matches. Five to six simultaneous loci matches are needed for A, B, and DRB1 matches, or at least 8/10 loci matches.

#### Algorithm for CB

The TNC in CB is limited and should be considered based on HLA-typing, MNC, and primary disease. For malignant hematological diseases, ≥ 4/6 loci should be matched, with TNC > (2.5–4.0) × 10^7^/kg (recipient weight) and CD34+ cells > (1.2–2.0) × 10^5^/kg (recipient weight). For non-malignant hematological diseases, ≥ 5/6 loci should be matched, with TNC > 3.5 × 10^7^/kg (recipient weight), and CD34+ cells > (1.7 × 10^5^/kg (recipient weight).

#### General principle of mobilization

Granulocyte colony-stimulating factor (G-CSF; 5 mg/kg of body weight per day for 5 days) was used to mobilize the BM and/or PB. The target mononuclear cell count was 6 × 10^8^/kg of recipient weight. Unmanipulated BM (harvested on day 4 after G-CSF) and/or PB stem cells (harvested on days 4 and 5 after G-CSF) were infused into the recipient on the day of collection.

### Conditioning regimens

The myeloablative regimen (MAC) mBuCy regimen in MSD-HSCT and the mBuCy+ATG regimen in haplo-HSCT are the most popular in China and achieve remarkable results (Tables 3). RIC or intensified conditioning regimen is also used for subgroups of patients (Tables 4 and 5).

**Table 3:** Traditional and modified myeloablative (MA) regimens

MAC	Drug	Dose(total)	Schedule(d)	Donor type
Traditional				
Cy/TBI	Cy	120 mg/kg	− 6, − 5	Allo-HSCT
	f-TBI	12 ~ 14 Gy	− 3 ~ − 1	
Bu/Cy	Bu	16 mg/kg(po)or 12.8/kg(iv)	−7 ~ − 4	Allo-HSCT
	Cy	120 mg/kg	− 3, − 2	
Modified (PUPH)				
mBuCy	Hu	80 mg/kg, divided in twice	− 10	HLA-matched sibling HSCT
	Ara-C	2 g/m^2^	− 9	
	Bu	9.6 mg/kg(iv)	− 8 ~ − 6	
	Cy	3.6 g/m^2^	− 5, − 4	
	MeCCNU	250 mg/m^2^(po)	− 3	
mCy/TBI	Single TBI	770 cGy	− 6	HLA-matched sibling HSCT
	Cy	3.6 g/m^2^	− 5, -4	
	MeCCNU	250 mg/m^2^	− 3	
mBuCy+ATG	Ara-C	4~ 8 g/m^2^	− 10, − 9	URD, CBT, HID-HSCT
	Bu	9.6 mg/kg(iv)	− 8 ~ − 6	
	Cy	3.6 g/m^2^	− 5, − 4	
	ATG	10 mg/kg	−5 ~ − 2	
	Or ATG-F	40 mg/kg	−5 ~ − 2	
mCy/TBI + ATG	TBI	770 cGy	− 6	URD-HSCT, HID-HSCT
	Cy	3.6 g/m^2^	− 5, − 4	
	MeCCNU	250 mg/m^2^	−3	
	ATG	10 mg/kg	−5 ~ − 2	
	Or ATG-F	40 mg/kg	−5 ~ − 2	

*Cy* cyclophosphamide, *Bu* busulfan, *TBI* total body irradiation, *Hu* hydroxyurea, *Ara-C* cytarabine, *MeCCNU* methyl CCNU, *ATG* anti-thymocyte globulin thymoglobuline, *ATG-F* rabbit anti-thymocyte globulin produced by Fresenius, *allo-HSCT* allogeneic hematological stem cell transplantation, *URD* unrelated donor, *CBT* cord blood transplantation, *HID-HSCT* haploidentical transplantation. *PUPH* Peking University People Hospital

**Table 4:** Reduced-intensity regimens for leukemia/myelodysplastic syndrome

Conditioning regimen	Drug	Dose (total)	Schedule(d)	Donor type
International regimen				
Flu/Mel	Flu	150 mg/m^2^	− 7 ~ − 3	Allo-HSCT
	Mel	140 mg/m^2^	− 2, − 1	
Flu/Bu	Flu	150 mg/m^2^	− 9 ~ − 5	Allo-HSCT
	Bu	8~ 10 mg/kg (po)	− 6 ~ − 4	
Flu/Cy	Flu	150 mg/m^2^	− 7 ~ − 3	Allo-HSCT
	Cy	140 mg/m^2^	− 2, − 1	
Flu/Bu/TT	Flu	150 mg/m^2^	− 7 ~ − 5	Allo-HSCT
	Bu	8 mg/kg (po)	− 6 ~ − 4	
	Thiotepa	5 mg/kg	− 3	
TBI/Cy/ATG	TBI	4 Gy	− 5	Flu+Ara-C+AMSA, followed by allo-HSCT
	Cy	120 mg/kg	− 4, − 3	
	ATG			
Modified regimen(PUPH)				
RIC-mBuCy	Hu	80 mg/kg (divided in two)	− 10	HLA-matched sibling HSCT
	Ara-C	2 g/m^2^ (CI)	− 9	
	Bu	4.8 mg/kg (iv)	− 10, − 9	
	Cy	2.0 g/m^2^	− 5, − 4	
	MeCCNU	250 mg/m^2^ (po)	− 3	
	ATG	10 mg/m^2^	− 5 ~ − 2	
	or ATG-F	40 mg/kg	− 5 ~ − 2	
RIC-BuFlu	Hu	80 mg/kg (divided in two)	− 10	HLA-matched sibling HSCT
	Ara-C	2 g/m2 (CI)	− 9	
	Bu	9.6 mg/kg (iv)	− 8 ~ − 6	
	Flu	150 mg/m^2^	− 6 ~ − 2	
	MeCCNU	250 mg/m^2^	− 3	
RIC-mBuFluATG	Ara-C	8 g/m^2^ (CI)	− 10/− 9	HID-HSCT
	Bu	9.6 mg/kg (iv)	− 8 ~ − 6	
	Flu	150 mg/m^2^	− 6 ~ − 2	
	MeCCNU	250 mg/m^2^	− 3	
	ATG	10 mg/kg	− 5 ~ − 2	
	or ATG-F	40 mg/kg	− 5 ~ − 2	
RIC-mBuCyFlu+ATG	Ara-C	8 g/m^2^ (CI)	− 10/− 9	HID-HSCT
	Bu	9.6 mg/kg (iv)	− 8 ~ − 6	
	Flu	150 mg/m^2^	− 6 ~ − 2	
	Cy	2.0 g/m^2^	− 5, − 4	
	MeCCNU	250 mg/m^2^	− 3	
	ATG	10 mg/kg	− 5 ~ − 2	
	or ATG-F	40 mg/kg	− 5 ~ − 2	

*Flu* fludarabine, *Mel* melphan, *Cy* cyclophosphamide, *Bu* busulfan, *TBI* total body irradiation, *Hu* hydroxyurea, *Ara-C* cytarabine, *MeCCNU* methyl CCNU, *ATG* anti-thymocyte globulin thymoglobuline, *ATG-F* rabbit anti-thymocyte globulin produced by Fresenius, *AMSA* amsacrine, *allo-HSCT* allogeneic hematological stem cell transplantation, *URD* unrelated donor, *CBT* cord blood transplantation, *HID-HSCT* haploidentical transplantation

**Table 5:** Intensified conditioning regimen

Conditioning regimen	Drugs	Dose(total)	Scheduled(d)	Donor type
International regimen				
Cy/VP/TBI	Cy	120 mg/kg	− 6, − 5	Allo-HSCT
	Vp16	30 ~ 60 mg/m^2^	− 4	
	FTBI	12.0 ~ 13.8Gy	− 3 ~ − 1	
TBI/TT/Cy	FTBI	13. 8 Gy	− 9 ~ − 6	Allo-HSCT
	TT	10 mg/kg (po)	− 5, − 4	
	Cy	120 mg/kg	− 6, − 5	
Bu/Cy/MEL	Bu	16 mg/kg (po)	− 7 ~ − 4	Allo-HSCT
	Cy	120 mg/kg	− 3, − 2	
	Mel	140 mg/m^2^	− 1	
Regimens in China				
Liu QF et al.	Flu	150 mg/m^2^	− 10 ~ − 6	Allo-HSCT
	Ara-C	5 ~ 10 g/m^2^	− 10 ~ − 6	
	TBI	9 Gy	− 5, − 4	
	Cy	120 mg/kg	− 3, − 2	
	Vp16	30 mg/kg	− 3, − 2	

*Cy* cyclophosphamide, *TT* thiotepa, *fTBI* fractional total body irradiation, *Flu*:fludarabine, *Bu* busulfan, *Mel* melphan, *Ara-C* cytarabine, *allo-HSCT* allogeneic hematological stem cell transplantation

Gao et al. retrospectively analyzed the outcomes of allo-SCT in 82 patients with AML or MDS who were conditioned with BuCy or fludarabine, idarubicin, intravenous-busulfan, and cytarabine (FIBA). There was no significant difference in the 3-year OS or the relapse rate, but RIC with FIBA exhibited a lower incidence of severe aGVHD and lower NRM than the BuCy regimen [83]. Intensified conditioning for patients with refractory leukemia, introduced by the Nanfang group, may reduce the high leukemia cell burden and improve outcomes. Lie et al. used a combination of fludarabine, cytarabine, TBI, Cy, and etoposide for conditioning in the haplo-setting and demonstrated that intensified conditioning decreased the 5-year relapse rate from 33.9 to 27.3%, and it may be a better approach for refractory and acute leukemia of ambiguous lineage [84–86]. Idarubicin-intensified haplo-HSCT introduced by the Wuhan Union group improved the dismal prognosis of pre-transplant MRD and yielded a 3-year DFS of 47.3% [87].

Selected older patients (age > 50) with low HCT-CI (≤ 2) and good performance status tolerate myeloablative HID-HSCT with similar outcomes compared to younger adults [57]. HID-HSCT with reduced-intensity regimens (RIC) substituted with cyclophosphamide with Flu was feasible in patients over 60 years of age and produced similar engraftment and relapse rates as myeloablative conditioning [29].

BuCy+ATG is a novel protocol developed and verified for HID-HSCT for SAA patients. It has been used for salvage therapy and first-line therapy in pediatric and adult patients [32–34].

### Recommendation: conditioning regimen

The definitions of MAC regimens and RIC are in accordance with EBMT [88].

1. MAC regimens
  - ≥ 100 mg/kg or 3.6 mg/m^2^ IV cyclophosphamide.
  - ≥ 12 Gy TBI
  - ≥ 16 mg/kg PO busulfan or 9.6 mg/kg IV busulfan
2. RIC regimens
  - 90–160 mg/m^2^ IV fludarabine
  - 6–9 mg/kg oral busulfan (or equivalent dose of IV busulfan)
  - 2–8 Gy TBI
  - 80–140 mg/m^2^ IV melphalan
  - 5–10 mg/kg IV thiotepa

### For patients with malignant hematological diseases

1. For patients with leukemia/MDS:

① Standard-intensity conditioning: MAC regimens include traditional TBICy, BuCy, and its modified regimens, and ATG is used in alternative donor transplantations at different doses. ATG (Thymoglobuline, Sanofi-Genzyme, Lyon, France) is used in a dose range of 6–10 mg/kg, and ATG-F (Grafalon, Neovii, Bad Homburg, Germany) is used in dose range of 20–40 mg/kg. ATG was recently used in MSD-HSCT to prevent GVHD. ② RIC: Fludarabine-containing regimens or ATG-included RIC regimens are used commonly (listed in Table 4). ③ Intensified regimens: Intensified regimens generally include the addition of a drug, such as Ara-C, VP16, Melphalan, TBI, Fludarabine, or Tespamin, to standard condition regimens. It is primarily used for refractory and relapsed malignant patients (Table 5).

The optimal conditioning regimen for a patient should be selected based on the type and status of the disease, comorbidities, underlying conditions, and donor source. For example, a standard-intensity conditioning regimen is used for younger patients (younger than 55 years old), and RIC regimens are used for patients older than 55 years of age and patients with poor organ function or HSCT-CI ≥ 3, regardless of age. Intensified regimens are used for young patients with refractory and relapsed diseases. Intensified regimens may reduce the incidence of relapse to some extent, but they often increase TRM. Therefore, these regimens may not significantly increase overall survival. RIC regimens are often better tolerated but require immunosuppression agents and cell therapy to reduce the relapse risk. Therefore, RIC regimens are combined with other regimens, such as fludarabine. HSCT with standard intensification conditioning regimen followed by immunosuppression adjustment or cell therapy to enhance the graft versus leukemia (GVL) effect is also feasible for refractory and relapsed patients.

2. For patients with malignant hematological diseases other than leukemia/MDS: Conditioning protocols, such as BEAM or Flu/Mel or Flu/Bu, are generally used for patients with MM or NHL (listed in Table 6). Myeloablative (MA) regimens of leukemia may also be used for patients with MM or NLH, such as BuCy, TBICy, or the modified BuCy regimen adopted by the Peking University People’s Hospital.

**Table 6:** Conditioning regimens for multiple myeloma and lymphoma

Conditioning regimen	Drug	Dose (total)	Time(d)	Transplantation type
BEAM	BCNU	300 mg/m^2^	−6	Allo-HSCT of lymphoma
	Vp16	800 mg/m^2^	−5 ~ − 2	
	Ara-C	800 mg/kg	−5 ~ − 2	
	Mel	140 mg/m^2^	−1	
Flu/MEL	Flu	150 mg/m2	−7 ~ − 3	Allo-HSCT of multiple myeloma
	Mel	140 mg/m^2^	−2, − 1	
	Bortizomib			
Flu/Bu	Flu	150 mg/m^2^	−10 ~ 6	Allo-HSCT of multiple myeloma
	Bu	6.4~ 9.6 mg/kg (iv)	− 7 ~ − 4	

*BCNU* carmustine, *VP16* etoposide, *Ara-C* cytarabine, *Mel* melphan, *Flu* fludarabine, *Bu* busulfan, *allo-HSCT* allogeneic hematological stem cell transplantation

### For patients with non-malignant hematological diseases

1. For patients with SAA: The Cy-ATG regimen is used for HLA-matched sibling transplantation, and the FluCy-ATG regimen is used for unrelated transplantations. There is no standard preparative regimen of choice for haploidentical transplantations. The most commonly used regimen in China is the BuCyATG protocol followed by T-replicate HID-HSCT (Table 7).
2. For patients with thalassemia: Intensified conditioning regimens instead of standard conditioning regimens for leukemia are generally used in patients with thalassemia (Table 8).
3. Fanconi anemia: The FluCyATG regimen (Flu 150 mg/m^2^, Cy 5–20 mg/kg/d × 4 days, and rabbit ATG 10 mg/kg) with or without low-dose TBI may be used for alternative donor transplantations.

**Table 7:** Conditioning regimens for severe aplastic anemia

Conditioning regimen	Drug	Dose (total)	Time(d)	Transplantation type
International regimen				
Cy-ATG	Cy	200 mg/kg	−5 ~ − 22	HLA-matched sibling HSCT
	ATG	11.25 ~ 15.00 mg/kg	−5 ~ − 3, − 2	
FluCy-ATG	Flu	120 mg/m^2^	−5 ~ − 2	HLA-matched unrelated HSCT
	Cy	120 mg/kg	−5, − 2	
	ATG	11.25 ~ 15.00 mg/kg	−5 ~ − 3, − 2	
Regimens in China				
mBuCyATG-SAA	Bu	6.4 mg/kg(ivgtt)	−7, − 6	Haploidentical-HSCT
	Cy	200 mg/kg	−5, − 2	
	ATG	10 mg/kg	−5 ~ − 2	
	or ATG-F	40 mg/kg	−5 ~ − 2	
mBuCyFluATG	Bu	6,4mg/kg(ivgtt)	−7 ~ − 6	Haploidentical-HSCT
	Flu	120 mg/m^2^	−10 ~ − 7	
	Cy	200 mg/kg	−6 ~ − 3	
	ATG-F	20 mg/kg	−4 ~ − 1	
	or ATG	10 mg/kg	−4 ~ − 1	
FluCy-ATG	Flu	120 mg/m^2^	−5 ~ − 2	Haploidentical-HSCT
	Cy	90 mg/kg	−3, − 2	
	ATG	10 mg/kg	−5 ~ − 2	

*Cy* cyclophosphamide, *ATG* anti-thymocyte globulin thymoglobuline, *ATG-F* rabbit anti-thymocyte globulin produced by Fresenius, *Flu* fludarabine, *Bu* busulfan, *allo-HSCT* allogeneic hematological stem cell transplantation

**Table 8:** Conditioning regimens for thalassemia

Conditioning regimen	Drug	Dose	Time(d)	Transplantation
General intensity				
Regimens for leukemia	The same as leukemia	Same as leukemia		Allo-HSCT
BuCy	Bu	14 mg/kg(po)		Allo-HSCT
	Cy	200 mg/kg		
Intensified regimen				
NF0-8-TM	Cy	110 mg/kg	−10, −9	HLA-matched sibling transplantation
	Flu	200 mg/m^2^	−8 ~ − 4	Unrelated HLA-matched transplantation
	TT	10 mg/kg	−5	
	Bu	ivgtt,QD(−8d) Css 300 ~ 600 ng/L	−8 ~ − 6	Haploidentical-HSCT
	Azathioprine	3 mg/kg,QD	From D−45	
	Hu	30 mg/kg,QD	From D−45	
FluBuCyATG	Flu	150mg/m^2^	−12 ~ − 10	Allo-HSCT
	Bu	12.8 mg/kg(ivgtt)	−9 ~ − 6	
	Cy	200 mg/kg	−5 ~ − 2	
	ATG	10 mg/kg	−5 ~ − 2	
	Hu	20 mg/kg,QD	3 months before	

*Bu* busulfan, *Cy* cyclophosphamide, *Flu* fludarabine, *TT* thiotepa, *ATG* anti-thymocyte globulin, *allo-HSCT* allogeneic hematological stem cell transplantation, *Css* steady state plasma concentration, *Hu* hydroxyurea, *QD* once a day

## Conclusion and perspective

In conclusion, this consensus is based on the standard of care and available clinical evidence in China. However, we recognize the limitation that some developmental indications and treatment options are also included because prospective clinical studies demonstrate that allo-HSCT is a promising treatment option compared to other non-HSCT strategies. Randomized prospective controlled trials are absent in most conditions because transplant decisions are complex issues. We recognize the need to periodically update these recommendations to keep abreast with ongoing research. In summary, we hope these recommendations developed by Chinese doctors inspire the refining of global clinical practice.

## Abbreviations

aGVHD: Acute graft-versus-host disease
allo-HSCT: Allogeneic hematopoietic stem cell transplantation
AP: Accelerated phase
ASBMT: American Society for Blood and Marrow Transplantation
ATG: Anti-thymocyte globulin
BC: Blastic crisis
BCSH: British Committee for Standards in Haematology
CB: Cord blood
CBMTR: Chinese Blood and Marrow Transplantation Registry
DSAs: Donor-specific anti-HLA antibodies
EBMT: European Group for Blood and Marrow Transplantation
FFS: Failure-free survival
GF: Primary graft failure
GVL: Graft versus leukemia
HID-HSCT: Haploidentical HSCT
HLA: Human leukocyte antigen
MAC: Myeloablative conditioning
MFI: Median fluorescence intensity
MMR: Major molecular remission
MRD: Minimal residual disease
MSD: HLA-matched sibling donor
MUD: HLA-matched unrelated donors
NCCN: National Comprehensive Cancer Network
NIMA: Non-inherited maternal antigen
NIPA: Non-inherited paternal antigen
PT-CY: Posttransplant cyclophosphamide
RIC: Reduced-intensity regimens
SAA: Severe aplastic anemia
TKIs: Tyrosine kinase inhibitors

## References

[1] Niederwieser D, Baldomero H, Szer J, Gratwohl M, Aljurf M, Atsuta Y, Bouzas LF, Confer D, Greinix H, Horowitz M (2016). Hematopoietic stem cell transplantation activity worldwide in 2012 and a SWOT analysis of the worldwide network for blood and marrow transplantation group including the global survey. Bone Marrow Transplant.

[2] Gratwohl A, Pasquini MC, Aljurf M, Atsuta Y, Baldomero H, Foeken L, Gratwohl M, Bouzas LF, Confer D, Frauendorfer K (2015). One million haemopoietic stem-cell transplants: a retrospective observational study. Lancet Haematol.

[3] Passweg JR, Baldomero H, Bader P, Bonini C, Duarte RF, Dufour C, Gennery A, Kroger N, Kuball J, Lanza F (2017). Use of haploidentical stem cell transplantation continues to increase: the 2015 European Society for Blood and Marrow Transplant activity survey report. Bone Marrow Transplant.

[4] Lv M, Huang X (2015). Fighting against hematological malignancy in China: from unique system to global impact. Sci China Life Sci.

[5] Xu LP, Wu DP, Han MZ, Huang H, Liu QF, Liu DH, Sun ZM, Xia LH, Chen J, Wang HX (2017). A review of hematopoietic cell transplantation in China: data and trends during 2008-2016. Bone Marrow Transplant.

[6] Du H, Chen J, Qin M, Fang J, Li Z, Zhu Y, Sun X, Huang D, Yu J, Tang Y (2015). Pediatric hematopoietic stem cell transplantation in China: data and trends during 1998–2012. Pediatr Transplant.

[7] Lee CJ, Savani BN, Mohty M, Labopin M, Ruggeri A, Schmid C, Baron F, Esteve J, Gorin NC, Giebel S (2017). Haploidentical hematopoietic cell transplantation for adult acute myeloid leukemia: a position statement from the Acute Leukemia Working Party of the European Society for Blood and Marrow Transplantation. Haematologica.

[8] Meng Lv YC, Xiaojun Huang: Everyone has a donor: contribution of the Chinese experience to global practice of haploidentical hematopoietic stem cell transplantation. Front Med:0-. 10.1007/s11684-017-0595-7 29675688

[9] Huang XJ, Han W, Xu LP, Chen YH, Liu DH, Lu J, Chen H, Zhang YC, Jiang Q, Liu KY, Lu DP (2004). A novel approach to human leukocyte antigen-mismatched transplantation in patients with malignant hematological disease. Chin Med J.

[10] Huang XJ, Liu DH, Liu KY, Xu LP, Chen H, Han W, Chen YH, Wang JZ, Gao ZY, Zhang YC (2006). Haploidentical hematopoietic stem cell transplantation without in vitro T-cell depletion for the treatment of hematological malignancies. Bone Marrow Transplant.

[11] Huang XJ, Liu DH, Liu KY, Xu LP, Chen H, Han W, Chen YH, Zhang XH, Lu DP (2009). Treatment of acute leukemia with unmanipulated HLA-mismatched/haploidentical blood and bone marrow transplantation. Biol Blood Marrow Transplant.

[12] Huang X, Liu D, Liu K, Xu L, Chen H, Han W, Chen Y, Wang Y, Zhang X (2009). Haploidentical hematopoietic stem cell transplantation without in vitro T cell depletion for treatment of hematologic malignancies in children. Biol Blood Marrow Transplant.

[13] Xiao-Jun H, Lan-Ping X, Kai-Yan L, Dai-Hong L, Yu W, Huan C, Yu-Hong C, Wei H, Jing-Zhi W, Yao C (2009). Partially matched related donor transplantation can achieve outcomes comparable with unrelated donor transplantation for patients with hematologic malignancies. Clin Cancer Res.

[14] Huang XJ, Chang YJ (2011). Unmanipulated HLA-mismatched/haploidentical blood and marrow hematopoietic stem cell transplantation. Biol Blood Marrow Transplant.

[15] Huang XJ, Zhu HH, Chang YJ, Xu LP, Liu DH, Zhang XH, Jiang B, Jiang Q, Jiang H, Chen YH (2012). The superiority of haploidentical related stem cell transplantation over chemotherapy alone as postremission treatment for patients with intermediate- or high-risk acute myeloid leukemia in first complete remission. Blood.

[16] Wang Y, Wu DP, Liu QF, Xu LP, Liu KY, Zhang XH, Xu Y, Huang F, Huang XJ (2018). Donor and recipient age, gender and ABO incompatibility regardless of donor source: validated criteria for donor selection for haematopoietic transplants. Leukemia.

[17] Wang Y, Liu QF, Xu LP, Liu KY, Zhang XH, Ma X, Wu MQ, Wu DP, Huang XJ (2016). Haploidentical versus matched-sibling transplant in adults with Philadelphia-negative high-risk acute lymphoblastic leukemia: a biologically phase III randomized study. Clin Cancer Res.

[18] Wang Y, Liu QF, Xu LP, Liu KY, Zhang XH, Ma X, Fan ZP, Wu DP, Huang XJ (2015). Haploidentical vs identical-sibling transplant for AML in remission: a multicenter, prospective study. Blood.

[19] Wang Y, Wang HX, Lai YR, Sun ZM, Wu DP, Jiang M, Liu DH, Xu KL, Liu QF, Liu L (2016). Haploidentical transplant for myelodysplastic syndrome: registry-based comparison with identical sibling transplant. Leukemia.

[20] Chen XH, Zhang C, Zhang X, Gao L, Gao L, Kong PY, Peng XG, Sun AH, Zeng DF, Wang QY (2010). Cost and outcome in stem cell collections in HLA-haplo identical/mismatched related transplantation with combined granulocyte-colony stimulating factor-mobilized blood and bone marrow for patients with hematologic malignancies. Transfus Apher Sci.

[21] Huang WR, Li HH, Gao CJ, Bo J, Li F, Dou LP, Wang LL, Jing Y, Wang L, Liu DH, Yu L (2016). Haploidentical, unmanipulated G-CSF-primed peripheral blood stem cell transplantation for high-risk hematologic malignancies: an update. Bone Marrow Transplant.

[22] Gao L, Wen Q, Chen X, Liu Y, Zhang C, Gao L, Kong P, Zhang Y, Li Y, Liu J (2014). Effects of priming with recombinant human granulocyte colony-stimulating factor on conditioning regimen for high-risk acute myeloid leukemia patients undergoing human leukocyte antigen-haploidentical hematopoietic stem cell transplantation: a multicenter randomized controlled study in southwest China. Biol Blood Marrow Transplant.

[23] Luo Y, Xiao H, Lai X, Shi J, Tan Y, He J, Xie W, Zheng W, Zhu Y, Ye X (2014). T-cell-replete haploidentical HSCT with low-dose anti-T-lymphocyte globulin compared with matched sibling HSCT and unrelated HSCT. Blood.

[24] Guo Z, Gao HY, Zhang TY, Liu XD, Yang K, Lou JX, He XP, Zhang Y, Chen P, Chen HR (2016). Analysis of allogeneic hematopoietic stem cell transplantation with high-dose cyclophosphamide-induced immune tolerance for severe aplastic anemia. Int J Hematol.

[25] Lv M, Huang XJ (2012). Allogeneic hematopoietic stem cell transplantation in China: where we are and where to go. J Hematol Oncol.

[26] Kasamon YL, Luznik L, Leffell MS, Kowalski J, Tsai HL, Bolanos-Meade J, Morris LE, Crilley PA, O'Donnell PV, Rossiter N (2010). Nonmyeloablative HLA-haploidentical bone marrow transplantation with high-dose posttransplantation cyclophosphamide: effect of HLA disparity on outcome. Biol Blood Marrow Transplant.

[27] Wang Y, Liu DH, Liu KY, Xu LP, Zhang XH, Han W, Chen H, Chen YH, Wang FR, Wang JZ (2013). Long-term follow-up of haploidentical hematopoietic stem cell transplantation without in vitro T cell depletion for the treatment of leukemia: nine years of experience at a single center. Cancer.

[28] Wang Y, Chang YJ, Xu LP, Liu KY, Liu DH, Zhang XH, Chen H, Han W, Chen YH, Wang FR (2014). Who is the best donor for a related HLA haplotype-mismatched transplant?. Blood.

[29] Sun YQ, Xu LP, Zhang XH, Liu DH, Chen H, Wang Y, Yan CH, Wang JZ, Wang FR, Zhang YY (2015). A retrospective comparison of BU-fludarabine and BU-CY regimens in elderly patients or in patients with comorbidities who received unmanipulated haploidentical hematopoietic SCT. Bone Marrow Transplant.

[30] Yu CL, Zheng D, Qiao ZH, Wang JM, Huang H, Liang YM, Wu DP, Chen BA, Bai H, Shi BF (2017). The long-term outcome of reduced-intensity allogeneic stem cell transplantation from a matched related or unrelated donor, or haploidentical family donor in patients with leukemia: a retrospective analysis of data from the China RIC Cooperative Group. Ann Hematol.

[31] Muffly L, Pasquini MC, Martens M, Brazauskas R, Zhu X, Adekola K, Aljurf M, Ballen KK, Bajel A, Baron F (2017). Increasing use of allogeneic hematopoietic cell transplantation in patients aged 70 years and older in the United States. Blood.

[32] Xu LP, Liu KY, Liu DH, Han W, Chen H, Chen YH, Zhang XH, Wang Y, Wang FR, Wang JZ, Huang XJ (2012). A novel protocol for haploidentical hematopoietic SCT without in vitro T-cell depletion in the treatment of severe acquired aplastic anemia. Bone Marrow Transplant.

[33] Xu LP, Wang SQ, Wu DP, Wang JM, Gao SJ, Jiang M, Wang CB, Zhang X, Liu QF, Xia LH (2016). Haplo-identical transplantation for acquired severe aplastic anaemia in a multicentre prospective study. Br J Haematol.

[34] Xu LP, Jin S, Wang SQ, Xia LH, Bai H, Gao SJ, Liu QF, Wang JM, Wang X, Jiang M (2017). Upfront haploidentical transplant for acquired severe aplastic anemia: registry-based comparison with matched related transplant. J Hematol Oncol.

[35] Zhu HH, Zhang XH, Qin YZ, Liu DH, Jiang H, Chen H, Jiang Q, Xu LP, Lu J, Han W (2013). MRD-directed risk stratification treatment may improve outcomes of t(8;21) AML in the first complete remission: results from the AML05 multicenter trial. Blood.

[36] Qin YZ, Xu LP, Chen H, Jiang Q, Wang Y, Jiang H, Zhang XH, Han W, Chen YH, Wang FR (2015). Allogeneic stem cell transplant may improve the outcome of adult patients with inv(16) acute myeloid leukemia in first complete remission with poor molecular responses to chemotherapy. Leuk Lymphoma.

[37] NCCN guidelines: Acute myeloid leukemia. Version 3.2017; http://www.nccn.org.

[38] Majhail NS, Farnia SH, Carpenter PA, Champlin RE, Crawford S, Marks DI, Omel JL, Orchard PJ, Palmer J, Saber W (2015). Indications for autologous and allogeneic hematopoietic cell transplantation: guidelines from the American Society for Blood and Marrow Transplantation. Biol Blood Marrow Transplant.

[39] Han LJ, Wang Y, Fan ZP, Huang F, Zhou J, Fu YW, Qu H, Xuan L, Xu N, Ye JY (2017). Haploidentical transplantation compared with matched sibling and unrelated donor transplantation for adults with standard-risk acute lymphoblastic leukaemia in first complete remission. Br J Haematol.

[40] NCCN guideline: Acute lymphoblastic leukemia. Version2.2017; http://www.nccn.org.

[41] Couban S, Savoie L, Mourad YA, Leber B, Minden M, Turner R, Palada V, Shehata N, Christofides A, Lachance S (2014). Evidence-based guidelines for the use of tyrosine kinase inhibitors in adults with Philadelphia chromosome-positive or BCR-ABL-positive acute lymphoblastic leukemia: a Canadian consensus. Curr Oncol.

[42] Chen H, Liu KY, Xu LP, Chen YH, Zhang XH, Wang Y, Qin YZ, Liu YR, Lai YY, Huang XJ (2017). Haploidentical hematopoietic stem cell transplantation for pediatric Philadelphia chromosome-positive acute lymphoblastic leukemia in the imatinib era. Leuk Res.

[43] Chen H, Liu KY, Xu LP, Chen YH, Han W, Zhang XH, Wang Y, Qin YZ, Liu YR, Huang XJ (2015). Haploidentical hematopoietic stem cell transplantation without in vitro T cell depletion for the treatment of philadelphia chromosome-positive acute lymphoblastic leukemia. Biol Blood Marrow Transplant.

[44] Gao L, Zhang C, Gao L, Liu Y, Su Y, Wang S, Li B, Yang T, Yuan Z, Zhang X (2015). Favorable outcome of haploidentical hematopoietic stem cell transplantation in Philadelphia chromosome-positive acute lymphoblastic leukemia: a multicenter study in Southwest China. J Hematol Oncol.

[45] Wang J, Jiang Q, Xu LP, Zhang XH, Chen H, Qin YZ, Ruan GR, Jiang H, Jia JS, Zhao T, et al. Allogeneic stem cell transplantation versus tyrosine kinase inhibitors combined with chemotherapy in patients with Philadelphia chromosome-positive acute lymphoblastic leukemia. Biol Blood Marrow Transplant. 2017. 10.1016/j.bbmt.2017.12.777. [Epub ahead of print]. 10.1016/j.bbmt.2017.12.777 29247779

[46] Xu ZL, Huang XJ, Liu KY, Chen H, Zhang XH, Han W, Chen YH, Wang FR, Wang JZ, Wang Y (2016). Haploidentical hematopoietic stem cell transplantation for paediatric high-risk T-cell acute lymphoblastic leukaemia. Pediatr Transplant.

[47] Chen Y, Cheng Y, Suo P, Yan C, Wang Y, Chen Y, Han W, Xu L, Zhang X, Liu K (2017). Donor-derived CD19-targeted T cell infusion induces minimal residual disease-negative remission in relapsed B-cell acute lymphoblastic leukaemia with no response to donor lymphocyte infusions after haploidentical haematopoietic stem cell transplantation. Br J Haematol.

[48] Jiang Q, Xu LP, Liu DH, Liu KY, Gale RP, Zhang MJ, Jiang B, Zhang XH, Wang Y, Chen SS (2013). Imatinib results in better outcomes than HLA-identical sibling transplants in young persons with newly diagnosed chronic-phase chronic myelogenous leukemia. Leukemia.

[49] Xu L, Zhu H, Hu J, Wu D, Jiang H, Jiang Q, Huang X (2015). Superiority of allogeneic hematopoietic stem cell transplantation to nilotinib and dasatinib for adult patients with chronic myelogenous leukemia in the accelerated phase. Front Med.

[50] Luo Y, Zhao Y, Tan Y, Shi J, Han X, Zheng Y, Li L, He J, Xie W, Ye X (2011). Imatinib combined with myeloablative allogeneic hematopoietic stem cell transplantation for advanced phases of chronic myeloid leukemia. Leuk Res.

[51] Jiang H, Xu LP, Liu DH, Liu KY, Chen SS, Jiang B, Jiang Q, Chen H, Chen YH, Han W (2014). Allogeneic hematopoietic SCT in combination with tyrosine kinase inhibitor treatment compared with TKI treatment alone in CML blast crisis. Bone Marrow Transplant.

[52] Xu LP, Xu ZL, Zhang XH, Chen H, Chen YH, Han W, Chen Y, Wang FR, Wang JZ, Wang Y (2016). Allogeneic stem cell transplantation for patients with T315I BCR-ABL mutated chronic myeloid leukemia. Biol Blood Marrow Transplant.

[53] Xu LP, Zhang XH, Wang FR, Mo XD, Han TT, Han W, Chen YH, Zhang YY, Wang JZ, Yan CH (2017). Haploidentical transplantation for pediatric patients with acquired severe aplastic anemia. Bone Marrow Transplant.

[54] Mo XD, Xu LP, Liu DH, Zhang XH, Chen H, Chen YH, Han W, Wang Y, Wang FR, Wang JZ (2013). The hematopoietic cell transplantation-specific comorbidity index (HCT-CI) is an outcome predictor for partially matched related donor transplantation. Am J Hematol.

[55] Chang YJ, Wang HT, Xu LP, Wang Y, Liu KY, Zhang XH, Liu DH, Chen H, Chen YH, Wang FR (2016). Combined model of the EBMT score modified model and the HCT-CI improves the stratification of high-risk patients undergoing unmanipulated haploidentical blood and marrow transplantation. Leuk Lymphoma.

[56] Wang HT, Chang YJ, Xu LP, Liu DH, Wang Y, Liu KY, Huang XJ (2014). EBMT risk score can predict the outcome of leukaemia after unmanipulated haploidentical blood and marrow transplantation. Bone Marrow Transplant.

[57] Chen Y, Wang Y, Xu LP, Liu KY, Chen H, Chen YH, Zhang XH, Wang FR, Han W, Wang JZ (2015). Haploidentical stem cell transplantation in patients aged 50 yr and older with leukemia: similar outcomes compared to younger adults. Clin Transpl.

[58] Yan CH, Jiang Q, Wang J, Xu LP, Liu DH, Jiang H, Chen H, Zhang XH, Liu KY, Huang XJ (2014). Superior survival of unmanipulated haploidentical hematopoietic stem cell transplantation compared with chemotherapy alone used as post-remission therapy in adults with standard-risk acute lymphoblastic leukemia in first complete remission. Biol Blood Marrow Transplant.

[59] Wang Y, Liu DH, Xu LP, Liu KY, Chen H, Chen YH, Han W, Zhang XH, Huang XJ (2012). Haploidentical/mismatched hematopoietic stem cell transplantation without in vitro T cell depletion for T cell acute lymphoblastic leukemia. Biol Blood Marrow Transplant.

[60] Sun YQ, Wang J, Jiang Q, Xu LP, Liu DH, Zhang XH, Liu KY, Huang XJ (2015). Haploidentical hematopoietic SCT may be superior to conventional consolidation/maintenance chemotherapy as post-remission therapy for high-risk adult ALL. Bone Marrow Transplant.

[61] Liu DH, Xu LP, Liu KY, Wang Y, Chen H, Han W, Zhang XH, Yan CH, Zhang YY, Wang JZ (2013). Long-term outcomes of unmanipulated haploidentical HSCT for paediatric patients with acute leukaemia. Bone Marrow Transplant.

[62] NCCN guideline :chronic myeloid leukemia. Version 1.2018; http://www.nccn.org.

[63] Jiang Q, Xu LP, Liu DH, Liu KY, Chen SS, Jiang B, Jiang H, Chen H, Chen YH, Han W (2011). Imatinib mesylate versus allogeneic hematopoietic stem cell transplantation for patients with chronic myelogenous leukemia in the accelerated phase. Blood.

[64] Ma YR, Huang XJ, Xu ZL, Liu KY, Chen H, Zhang XH, Han W, Chen YH, Wang FR, Wang JZ (2016). Transplantation from haploidentical donor is not inferior to that from identical sibling donor for patients with chronic myeloid leukemia in blast crisis or chronic phase from blast crisis. Clin Transpl.

[65] NCCN guideline :MPN. Version 1.2018; http://www.nccn.org.

[66] Chen Y, Liu K, Xu L, Chen H, Liu D, Zhang X, Shi H, Han W, Wang Y, Zhao T (2010). HLA-mismatched hematopoietic SCT without in vitro T-cell depletion for myelodysplastic syndrome. Bone Marrow Transplant.

[67] NCCN guideline :multiple myeloma. Version 3.2017; http://www.nccn.org.

[68] NCCN guideline :HD. Version 1.2017; http://www.nccn.org.

[69] NCCN guideline :NHL. Version 1.2017; http://www.nccn.org.

[70] Gao L, Li Y, Zhang Y, Chen X, Gao L, Zhang C, Liu Y, Kong P, Wang Q, Su Y (2014). Long-term outcome of HLA-haploidentical hematopoietic SCT without in vitro T-cell depletion for adult severe aplastic anemia after modified conditioning and supportive therapy. Bone Marrow Transplant.

[71] Chang YJ, Luznik L, Fuchs EJ, Huang XJ (2016). How do we choose the best donor for T-cell-replete, HLA-haploidentical transplantation?. J Hematol Oncol.

[72] Chang YJ, Zhao XY, Xu LP, Zhang XH, Wang Y, Han W, Chen H, Wang FR, Mo XD, Zhang YY (2015). Donor-specific anti-human leukocyte antigen antibodies were associated with primary graft failure after unmanipulated haploidentical blood and marrow transplantation: a prospective study with randomly assigned training and validation sets. J Hematol Oncol.

[73] Zhao XY, Chang YJ, Xu LP, Zhang XH, Liu KY, Li D, Huang XJ (2014). HLA and KIR genotyping correlates with relapse after T-cell-replete haploidentical transplantation in chronic myeloid leukaemia patients. Br J Cancer.

[74] Zhao XY, Huang XJ, Liu KY, Xu LP, Liu DH (2007). Prognosis after unmanipulated HLA-haploidentical blood and marrow transplantation is correlated to the numbers of KIR ligands in recipients. Eur J Haematol.

[75] Zhao XY, Chang YJ, Zhao XS, Xu LP, Zhang XH, Liu KY, Li D, Huang XJ (2015). Recipient expression of ligands for donor inhibitory KIRs enhances NK-cell function to control leukemic relapse after haploidentical transplantation. Eur J Immunol.

[76] Huang XJ, Zhao XY, Liu DH, Liu KY, Xu LP (2007). Deleterious effects of KIR ligand incompatibility on clinical outcomes in haploidentical hematopoietic stem cell transplantation without in vitro T-cell depletion. Leukemia.

[77] Long H, Lu ZG, Song CY, Huang YX, Xu JH, Xu JX, Deng L, Tu SF, He YZ, Lin X (2016). Long-term outcomes of HLA-haploidentical stem cell transplantation based on an FBCA conditioning regimen compared with those of HLA-identical sibling stem cell transplantation for haematologic malignancies. Bone Marrow Transplant.

[78] Zhao X, Gao F, Zhang X, Wang Y, Xu L, Liu K, Zhao X, Chang Y, Wei H, Chen H (2016). Improved clinical outcomes of rhG-CSF-mobilized blood and marrow haploidentical transplantation compared to propensity score-matched rhG-CSF-primed peripheral blood stem cell haploidentical transplantation: a multicenter study. Sci China Life Sci.

[79] Sun Y, Beohou E, Labopin M, Volin L, Milpied N, Yakoub-Agha I, Piemontese S, Polge E, Houhou M, Huang XJ (2016). Unmanipulated haploidentical versus matched unrelated donor allogeneic stem cell transplantation in adult patients with acute myelogenous leukemia in first remission: a retrospective pair-matched comparative study of the Beijing approach with the EBMT database. Haematologica.

[80] Gragert L, Eapen M, Williams E, Freeman J, Spellman S, Baitty R, Hartzman R, Rizzo J, Horowitz M, Confer D, Maiers M (2014). HLA match likelihoods for hematopoietic stem-cell grafts in the U.S. registry. N Engl J Med.

[81] Mo XD, Tang BL, Zhang XH, Zheng CC, Xu LP, Zhu XY, Wang Y, Liu HL, Yan CH, Chu XD (2016). Comparison of outcomes after umbilical cord blood and unmanipulated haploidentical hematopoietic stem cell transplantation in children with high-risk acute lymphoblastic leukemia. Int J Cancer.

[82] Chang YJ, Wang Y, Liu YR, Xu LP, Zhang XH, Chen H, Chen YH, Wang FR, Han W, Sun YQ (2017). Haploidentical allograft is superior to matched sibling donor allograft in eradicating pre-transplantation minimal residual disease of AML patients as determined by multiparameter flow cytometry: a retrospective and prospective analysis. J Hematol Oncol.

[83] Gao L, Gao L, Gong Y, Zhang C, Chen XH, Zhang X (2013). Reduced-intensity conditioning therapy with fludarabine, idarubicin, busulfan and cytarabine for allogeneic hematopoietic stem cell transplantation in acute myeloid leukemia and myelodysplastic syndrome. Leuk Res.

[84] Liu QF, Fan ZP, Zhang Y, Jiang ZJ, Wang CY, Xu D, Sun J, Xiao Y, Tan H (2009). Sequential intensified conditioning and tapering of prophylactic immunosuppressants for graft-versus-host disease in allogeneic hematopoietic stem cell transplantation for refractory leukemia. Biol Blood Marrow Transplant.

[85] Liu QF, Fan ZP, Wu MQ, Sun J, Wu XL, Xu D, Jiang QL, Zhang Y, Huang F, Wei YQ (2013). Allo-HSCT for acute leukemia of ambiguous lineage in adults: the comparison between standard conditioning and intensified conditioning regimens. Ann Hematol.

[86] Xuan L, Fan Z, Zhang Y, Zhou H, Huang F, Dai M, Nie D, Lin D, Xu N, Guo X (2016). Sequential intensified conditioning followed by prophylactic DLI could reduce relapse of refractory acute leukemia after allo-HSCT. Oncotarget.

[87] Zhang R, Shi W, Wang HF, You Y, Zhong ZD, Li WM, Zhang C, Lu X, Wang YD, Zheng P (2017). Idarubicin-intensified haploidentical HSCT with GvHD prophylaxis of ATG and basiliximab provides comparable results to sibling donors in high-risk acute leukemia. Bone Marrow Transplant.

[88] Rubio MT, Savani BN, Labopin M, Piemontese S, Polge E, Ciceri F, Bacigalupo A, Arcese W, Koc Y, Beelen D (2016). Impact of conditioning intensity in T-replete haplo-identical stem cell transplantation for acute leukemia: a report from the acute leukemia working party of the EBMT. J Hematol Oncol.

